# Supplementary material for: Formulation of Lipid-Based Nanoparticles for Simultaneous Delivery of Lapatinib and Anti-Survivin siRNA for HER2+ Breast Cancer Treatment
Source: Pharmaceuticals (Basel). 2022 Nov 23;15(12):1452. doi: 10.3390/ph15121452 (PMC9784347; doi:10.3390/ph15121452)
Supplement: Supplementary file 1 [file pharmaceuticals-15-01452-s001.zip › pharmaceuticals-1947900-supplementary.pdf]

pharmaceuticals

MDPI

Supplementary data

S1: Storage physicochemical stability of the LBNP

The LBNP with three different chitosan concentrations (1×, 2×, and 3×, respectively) were stored at a low temperature (4°C) and protected from light. On days 0, 7, 14, 21, and 28, particle size, PDI, and zeta potential (Fig. S1) were measured for each formulation after siRNA addition on the selected day; also, gel retardation assays were performed in parallel (Fig. 2 and S2). These experiments were performed to investigate the stability of LBNP with various chitosan concentrations over time and to select the optimum chitosan concentration for other nanoparticles' physicochemical and cellular activity evaluations.

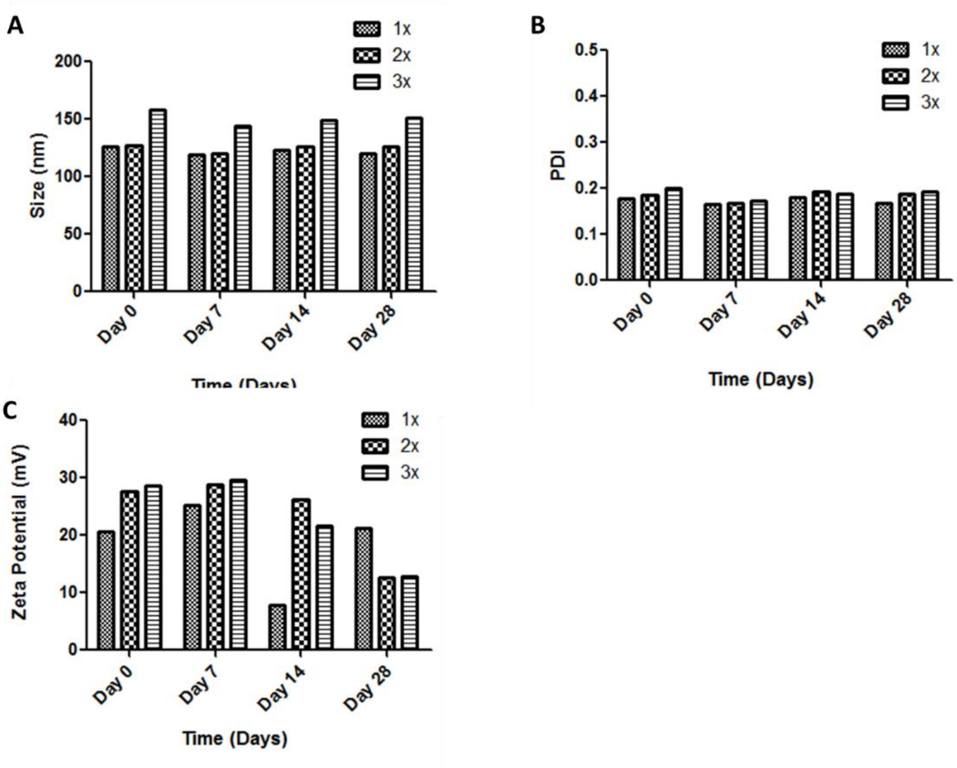

Figure S1. The graphs represented size (A), PDI (B), and zeta potential (C) of the siRNA_LBNP with different chitosan concentrations on days: 0, 7, 14, and 28. The initial theoretical chitosan concentration (0.6mg/mL) is 1x, while the double and triple chitosan concentrations are 2x and 3x, respectively. The chitosan-modified LBNP storage temperature was 4°C and the ctrl. siRNA (2μM) was added at each time point before size, PDI, and zeta measurements.

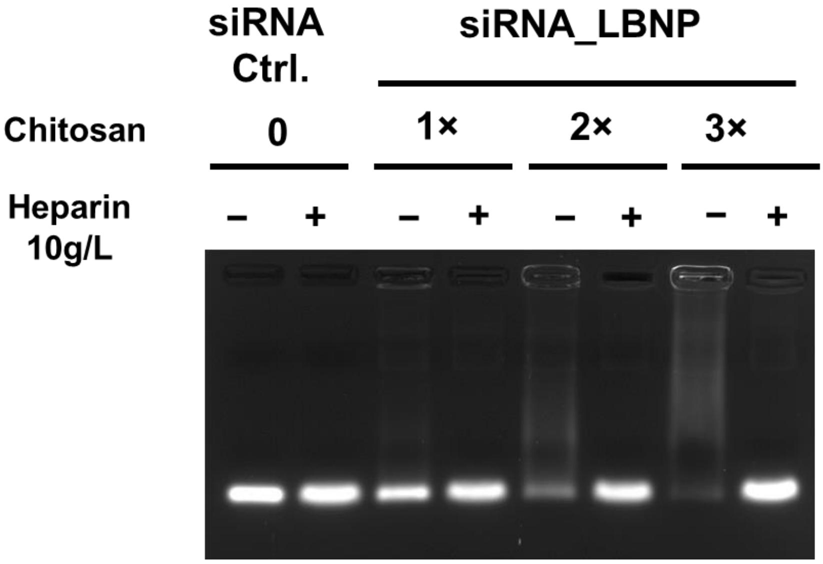

Figure S2. Gel retardation assay image demonstrating the siRNA protection in the modified lipid-based nanoparticles with different chitosan concentrations at day 28. The initial theoretical chitosan concentration of 0.6 mg/mL is referred to as 1x, while the double and triple of the initial chitosan concentrations are referred to as 2x and 3x, respectively. siRNA formulated in blank loaded LBNP in the presence (+) or absence (-) of heparin compared to naked siRNA. Lanes without heparin show free siRNA amount, and lanes with heparin show total siRNA amount in the sample. The siRNA was added just before the analysis by agarose gel elecrtophoresis.

S2: physicochemical characteristics of fluorescent LBNP and ATTO 488 siRNA labeled LBNP

Table S1: Size measurement of DiD/ATTO 488 siRNA_LBNP

Formulation	D_H (nm)	PDI	ζ (mV)
DiD_LNCs	91.3 ± 1.8	0.134 ± 0.0115	−1.91 ± 0.16
DiD_LBNP	130.2 ± 2.65	0.103 ± 0.013	3.74 ± 1.2
siCtrl.-DiD_LBNP	134.67 ± 11.64	0.118 ± 0.045	−0.33 ± 0.26
ATTO 488 siRNA_LBNP	143.1	0.154	-

D_H: Hydrodynamic size, **DiD:** DiIC18(5); 1,1′-dioctadecyl-3,3,3′,3′- tetramethylindodicarbocyanine, 4-chlorobenzenesulfonate salt **PDI:** Polydispersity index, **LNCS:** Lipid nanocapsules, **LBNP:** Lipid-based nanoparticles, **mV:** millivolt.

S3: siRNA transfection and western blot

3×10^5 cells/well were seeded in a 6-well plate for 24h on the day of transfection, anti-Survivin siRNA, or Ctrl. siRNA with blank or LAPA_LBNP were prepared and diluted in OptiMEM serum-free medium for 4h at 100nM. Afterward, the SK-BR-3 culture medium was added (1:1v/v), and the cells were maintained in normal growth conditions for an additional 68h (final siRNA concentration of 50nM). Cells were treated with the prepared suspensions and maintained in normal growth conditions for 72h. After incubating the different formulations for 72h, transfected cells were washed with cold PBS, and total proteins were extracted on ice by RIPA buffer supplemented with anti-protease (Sigma-Aldrich Chemie GmbH, Schnelldorf, Germany). After 15min centrifugation (10,000G, 4°C) to collect supernatant, protein concentrations were determined using a bicinchoninic acid (BCA) protein assay kit (Bio-rad, Hercules, CA). The cell lysate was heated up to 100°C for 7min in an SDS sample buffer and subjected to SDS-polyacrylamide gel electrophoresis (PAGE). The proteins are transferred to the nitrocellulose membrane using an iBlot® 2 dry blotting system (Thermo Fisher Scientific, Illkirch, France). Following membrane blocking with 5% nonfat milk at room

temperature for 1h, then the membrane was incubated with the primary antibody against Survivin (anti-rabbit, 1:1000, Life Technologies) at 4°C overnight and then with glyceraldehyde-3-phosphate dehydrogenase (GAPDH, anti-rabbit, 1:1000, Life Technologies) at room temperature for 2h. Afterward, the membrane was incubated with the peroxidase-conjugated secondary antibody (HRP goat to rabbit, 1:1000, Life technologies) for 1h. Between the incubations, the membrane was washed 3 times with Tris-buffered saline with 0.1% Tween® (TBST). The protein was visualized via an enhanced chemiluminescence (ECL) kit (Thermo Pierce) on a Fusion-Solo. 65.WL imager (Vilbert Lourmat, Marne-la Vallée, France) using EvolutionCapt software.

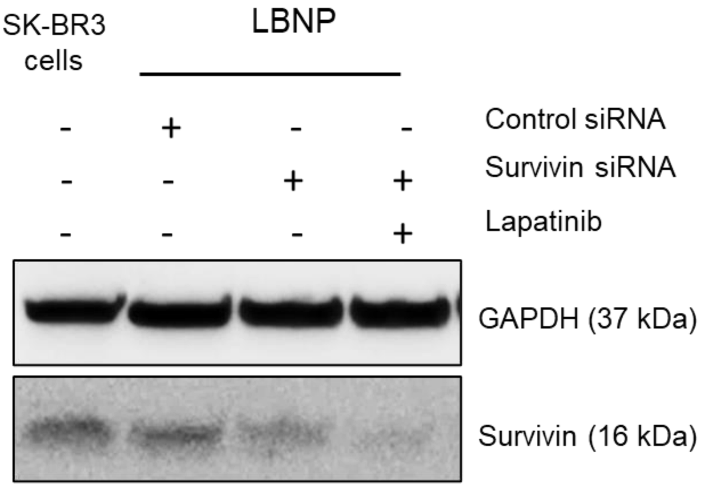

Figure S3. Down-regulation efficiency of modified lipid-based nanoparticles on Survivin protein expression. Western Blot of SK-BR-3 cells untreated (SK-BR-3 cells) or treated with LBNP loaded with or without LAPA and/or anti-Survivin siRNA/ control siRNA for 72h.
